# El acceso es esencial: procedural compliance alone does not ensure meaningful Latino parent participation in special education

**DOI:** 10.3389/fpsyg.2026.1815521

**Published:** 2026-07-13

**Authors:** Laura J. Malagon-Palacios, Kimber L. Bruner, Hayley M. Mullinax, Clarissa N. Sevillano, Erin Yosai

**Affiliations:** Department of Educational Psychology, University of Kansas, Lawrence, KS, United States

**Keywords:** culturally and linguistically diverse (CLD), educational equity, IDEA, language access, Latino families, parent engagement, school psychology, special education

## Abstract

Ensuring equitable family participation is a foundational requirement of special education policy in the United States, yet persistent disparities indicate that access is not experienced uniformly across populations. This practice and policy review synthesizes interdisciplinary research on Latino parents’ engagement in Child Find and Individualized Education Program (IEP) processes under the Individuals with Disabilities Education Act (IDEA), with emphasis on families who prefer Spanish or have limited English proficiency. Findings across legal, educational, and psychological literature identify converging barriers, including limited language access, inconsistent interpretation and translation practices, culturally incongruent communication, and immigration-related concerns that may inhibit trust and participation. These factors can compromise informed consent and reduce the effectiveness of family–school collaboration. The review analyzes how IDEA procedural safeguards interact with federal civil rights guidance and professional standards in school psychology, revealing gaps driven by ambiguous policy language and variable implementation. Implications highlight multilevel strategies—culturally responsive practices, structured parent supports, and systems-level consultation—to advance equitable access and strengthen outcomes for culturally and linguistically diverse students with disabilities.

## Introduction

In the fall of 2022, Latino^1^ students made up 29% of the total U. S. public school population (National Center for Education Statistics, 2024) and accounted for approximately 15% of students receiving special education and related services under the Individuals with Disabilities Education Act (IDEA; National Center for Education Statistics, 2023). These statistics indicate that Latino students are the second largest racial group represented under IDEA even though there is mixed evidence that Latino students experience both over- and under-identification for special education eligibility depending on factors such as geographic location, language proficiency, and assessment practices (Guiberson, 2009; Artiles et al., 2010).

Students are identified for special education services through the Child Find process, which may ultimately result in the decision to place a student on an Individualized Education Program (IEP) process. The Child Find process is a federal process under the IDEA (34 C.F.R. § 300.111; U.S. Department of Education, 2017) that mandates the “Zero Reject Principle,” meaning that schools must actively locate, accurately identify, and efficiently evaluate children who may have disabilities, even if the child is not yet failing or formally referred, to ensure a free and appropriate public education (FAPE).

Child Find is intended for children ages 3–21 and mandates that all public schools, private schools, homeschools, and preschools participate. Children may be identified through academic difficulties, behavioral or emotional difficulties, medical diagnoses, screening results, response to intervention data, teacher concerns, and, of equal importance, parent concerns. The Child Find team is made up of various professionals (e.g., school psychologists, special education teachers, etc.) including the Local Education Agency (LEA), which is the public board of education that maintains administrative control of public elementary or secondary schools, and the child’s parents. The process begins with a Child Find referral, parental consent, and a comprehensive assessment, which is followed by an IEP meeting to determine eligibility, goals, and services. The results are then discussed at the eligibility meeting where goals, services, and accommodations are outlined if the student qualifies. Parents must be provided with a Prior Written Notice (PWN), the district’s outlined plan to evaluate IEP student placement, and with a Procedural Safeguard Notice, which outlines a parent’s rights under IDEA (Individuals with Disabilities Education Act [IDEA], 2004).

Although the IEP process is designed to ensure equitable access, systemic inequalities continue to affect how Latino/Hispanic families are engaged and informed. For Latino families, participation in the IEP process includes navigating legal knowledge while preserving cultural assets such as *familismo* (i.e., values that emphasize strong family support)*, collectivismo* (i.e., focus on community rather than the individual), religion or *spiritualismo* (e.g., Catholicism or indigenous rituals), *respeto* (i.e., maintaining respect for authority), *personalismo* (i.e., prioritizing interpersonal relationships over status or personal gain), *idioms of distress* (i.e., culturally specific ways of expressing psychological distress, such as *ataque de nervios*), and hierarchical parent structures (e.g., *marianismo*, reflecting expectations of mothers as a caregiver, and *machismo*, reflecting expectations of fathers as authority figures and providers; Banda et al., 2025; Cahill et al., 2021; Foxen, 2016; Ijadi-Maghsoodi et al., 2023). These cultural assets can support collaborative decision making and advocacy when they are integrated into school practices. However, while IDEA prioritizes procedural compliance, they provide limited guidance for practicing school psychologists on how to approach these procedures for culturally linguistic diverse (CLD) populations and particularly for families with limited English proficiency. As a result, these limited policy guidelines under IDEA place responsibilities on families to navigate complex systems, while school systems vary on how to implement best practices for CLD populations. Variation in state-wide and district-wide procedures, such as translation procedures, influence whether Latino cultural assets are supported through the Child Find process.

Additionally, practicing school psychologists are called to follow practice guidelines from the national governing and licensing agency, the National Association of School Psychologists (NASP; National Association of School Psychologists, 2026). NASP provides detailed information on best practice guidance and practical application suggestions for interpreting and navigating the IDEA legislation. For example, NASP’s Domain 3 provides ethical expectations that emphasize the responsibility of school psychologists to engage in culturally responsive and nondiscriminatory assessment practices (National Association of School Psychologists, 2020). As a result, the way these are operationalized may vary across school context based on demographics and resources. For example, Scott et al. (2014) found that majority of the states did not address culturally and linguistically diverse students beyond federal requirements, whereas a smaller number of states, such as Connecticut, provided more detailed guidance, including the use of native language assessment, professional development, keeping track of language development and incorporating bilingual evaluators. However, many of NASP’s detailed practice guidelines are housed behind a paid membership wall, which may limit practitioners’ access to valuable and ever-changing resources. Variability in access to professional resources and the complexity of IDEA’s policies may contribute to confusion among practitioners regarding how to best support CLD students and families.

Although federal statues such as IDEA mandate meaningful access and nondiscrimination, Latino families continue to encounter linguistic, cultural, and systemic barriers during the Child Find process. For this review, we primarily drew from recent studies examining Latino family experiences during the Child Find process, as well as the IDEA policy document to better understand why some Latino families experience gaps in services and how IDEA can be expanded to reduce these gaps. This practice and policy review examines how these barriers faced by Latino parents may influence their participation in the Child Find process. This review also explores how IDEA’s procedural protections can be improved to support culturally responsive practices that can help strengthen home-school collaboration, thereby improving IEP goal attainment for Latino students receiving special education. This call for reform is crucial for the field of school psychology, as the NASP outlines family, school, and community collaboration, as well as equitable practices for diverse student populations, as core ethical responsibilities in Domain 8 (National Association of School Psychologists, 2020).

## Barriers to participation

### Language and access

While the special education IEP process can be overwhelming for all parents, including English-only speaking families (Sanderson, 2023; Alba, 2025), Latino parents often encounter additional barriers related to language and access, cultural mismatch and trust, and other systemic factors. For example, a review by Alba (2025) analyzed 19 articles published between the years 2004 and 2024 that identified differences in IEP experiences between English-speaking families and Spanish-speaking families. This research reported that, across two decades, families continued to report confusion about the IEP process, regardless of language background; however, Spanish-speaking families experience additional barriers related to language access, which may limit their understanding of IEP procedures and advocacy during IEP meetings (Alba, 2025). These challenges may individually and collectively limit meaningful participation and underscore critical concerns about equity in special education practices and policy. As special education and related services can relate to a variety of educational services, including gifted education, career and technical programming, and dual-language programming, the issues of access within IEP participation negatively affect families and students with a range of needs, beyond the typical support afforded by special education resources.

To further illustrate these findings, Bravo-Ruiz and Flynn (2022) examined responses from 50 Latino parents regarding their involvement in the special education process and the relationship between parental engagement and contextual factors, including English language communication skills and length of residence in the United States. Parents who reported stronger English communication skills were more likely to participate actively in the Child Find process. However, Bravo-Ruiz and Flynn (2022) found that the length of time living in the United States was not a significant predictor of parental participation, suggesting that exposure to U.S. education system over time does not necessarily indicate greater parental participation or increased confidence. Importantly, their research showed that even parents with stronger English skills reported confusion related to special education procedures, suggesting that language proficiency alone does not eliminate barriers to meaningful participation. On the other hand, those parents with less proficient English skills were more uncomfortable asking questions, had difficulty understanding information, and often relied on translators (Bravo-Ruiz and Flynn, 2022). This demonstrates that, while parents may attempt to participate in IEP meetings, they can experience reduced confidence and confusion.

Similar patterns were observed in another comparative study examining the differences between English-speaking and Spanish-speaking families within the special education system (Mercado-Anazagasty et al., 2025). Authors of the study surveyed 177 parents of children with disabilities (*n* = 88 Spanish-speaking, *n* = 89 English-speaking; 64% identified as Latinx/Hispanic) on stress levels, knowledge of special education procedures, participation in IEP meetings, barriers to attendance, experiences of discrimination, parental satisfaction, and Spanish-speaking parents’ specific concerns. Results indicated that Latino parents with stronger English language skills were more likely to demonstrate greater knowledge of the IDEA and the special education evaluation process, attend IEP meetings, bring school advocates and actively participate in educational decision making compared to Latino parents with limited English proficiency (Mercado-Anazagasty et al., 2025). In tandem with the Bravo-Ruiz and Flynn (2022) study Mercado-Anazagasty et al. (2025) further emphasizes that English language skills can promote engagement in the IEP process for Spanish-speaking families, especially given the complexity of this process that is already difficult for families regardless of language proficiency (Alba, 2025).

To bridge this cultural gap, IDEA specifies the use of interpreters “when necessary” or to take “whatever action is necessary” to ensure parents have full understanding of the IEP process (Individuals with Disabilities Education Act Regulations (2026). 34 C.F.R. § 300.322(e)). However, IDEA policy lacks explicit guidance regarding the type of interpreter to be provided, such as whether interpreters must be professionally trained, fluent in special education terminology, or independent from school personnel, which could result in inconsistent and inequitable implementation across districts. In contrast, National Association of School Psychologists (2020) Standard II.3.9 emphasizes the professional responsibility to request interpreters who are “qualified” and acceptable to clients who are, in this case, the students’ parents. Even despite this ethical guidance, however, neither IDEA nor NASP provide concrete, systems-level direction regarding how schools or professionals should identify, train, or ensure consistent access to qualified interpreters. In fact, Weirick (2023) clarified that under IDEA, interpreters are recognized as professional staff and not “helpers,” meaning that interpreters are essential for service delivery and should be qualified.

Emerging research suggests that qualified translators are essential for preventing discriminative practices because poor translating services are linked to reduced school-family communication and reduce trustworthiness in partnership with the school (Yan et al., 2022). A recent case study examining interpretation practices found that interpreters were frequently asked to provide services beyond direct translation which included conveying tone and facial expressions. The study also noted that many interpreters were bilingual school staff rather than formally trained interpreters and were often expected to fulfill multiple roles during interactions with families (Batz and Santillán, 2025). Therefore, federal policy should provide more explicit guidance regarding interpreter qualifications, and role expectations within special education. Clearer policies could help ensure that interpreters are adequately prepared to support families throughout referral, evaluation, eligibility determination, and service delivery processes.

Similarly, many Spanish-speaking Latino parents report receiving meeting notices and IEP documents in English (Barrera-Lansford and Sánchez, 2024; Guerrero, 2023; Montoya et al., 2022) despite IDEA and NASP guidelines to support CLD families, which makes the special education process distinctly inhibitive to their ability to participate compared to English-speaking families. This practice of providing documents to make educational decisions that require parental consent in a language that Latino parents might not understand could increase the likelihood of many Latino families signing legal documents without fully understanding their content or consequences, which is a violation of federal education law, as well as an ethical crisis for the field of school psychology.

These trends were evident in a qualitative study using two semi-structured interviews with ten Hispanic mothers who had recently begun the IEP process (Guerrero, 2023). One interview focused on acquiring information about their positive and negative experiences during the process, while the second interview focused on how these mothers acquired IEP knowledge during the process. They found that mothers especially struggled with understanding special education processes when they did not receive IEP documents in their native language; in fact, only one mom was able to accurately name the disability diagnosis her child was receiving (Guerrero, 2023). This specific finding highlights a significant concern, as linguistic barriers, rather than parental capacity or engagement, may prevent parents from fully exercising their role in the Child Find process. In these cases, parental rights may be compromised in practice; not due to intentional actions by school staff, but because of unclear policy guidance.

Federal civil rights guidance under Title VI provides an additional layer of protection for families with limited English proficiency beyond the procedural requirements outlined in IDEA and the ethical standards articulated by NASP. Specifically, Title VI requires school districts to ensure meaningful access to programs and services by translating “vital documents” (U.S. Department of Education, Office for Civil Rights and U.S. Department of Justice, 2015). However, like IDEA, this federal guidance does not clearly define which special education documents qualify as “vital” beyond PWN and procedural safeguards (Individuals with Disabilities Education Act, 2004, 34C. F. R. §§ 300.503–300.504). While parents are entitled to receive information regarding PWN and procedural safeguards in their native language, federal law does not explicitly require translation of all other IEP-related documents. As a result, states and districts may limit translation practices to procedural compliance, leaving crucial IEP materials untranslated and, thus, inaccessible to Spanish-speaking families.

Although broad federal policies cannot specify responses to specific cultural groups, multiple professional frameworks demonstrate how cultural and linguistic diversity can be incorporated through putting ethical values into practice through legal advocacy. A few state departments of education have imposed additional requirements that extend beyond federal mandates. For example, the Colorado Department of Education requires translation of IEP documents and mandates that parents be verbally informed of their right to request translation services (Colorado Department of Education, 2023). In contrast, many states have not implemented similar requirements. A preliminary descriptive scan of publicly available state special education policies suggests that roughly two-thirds of states appear to only comply with what is written in the federal law. There are no specific policies within the state that schools follow to ensure parents are receiving proper documentation that they can understand. Because of this, responsibility falls on parents to request this resource from the school that they may or may not know about. On the other hand, one-third of the states have created their own state policies with more strict regulations on what is considered a vital document as well as overall access to translation services. For example, California Education Code (2019) § 48,985 mandates that schools with a population of 15% or more of non-English-speaking caregivers are provided with documents translated to their primary language. The documents specified are “all notices, reports, statements, or records sent to the parent or guardian of any such pupil by the public school or school district.” The remaining states encourage more regulation within their state, but it remains questionable about what counts as a vital document and leave it to the schools to decide.

Considering that some Spanish-speaking Hispanic parents report signing IEP documents while still feeling unsure about what the documents meant, language accessibility becomes central to whether informed consent is truly achieved (Montoya et al., 2022). The field of school psychology, which is a major stakeholder in special education and related services, outlines in standard I.1.3 parental informed consent as an ethical and legal imperative when conducting special education evaluation and re-evaluation, as well as when creating and updating student IEPs (National Association of School Psychologists, 2020). Signatures alone are not sufficient; if parents cannot understand what they are signing, informed consent has not been achieved. Thus, current research findings introduce the need for the review and implementation of consistent state and district policies that mandate both the translation of “vital documents” and guarantee access to information about special education and related services for LEP parents.

Meaningful parental participation is essential for accurate special education classification and support. However, barriers such as untranslated documents or a lack of culturally responsive engagement from members of the school may prevent Latino families from being full partners in their children’s special education and IEP processes. Although many parents express a strong desire to participate in the IEP process and better understand their rights under IDEA (Barrera-Lansford and Sánchez, 2024), many report limited access to culturally and linguistically appropriate resources. These missed opportunities for collaboration risk inappropriate services, misdiagnoses, IEP goal attainment, and increased dissatisfaction.

### Cultural mismatch and trust

Research indicates that cultural mismatch and lack of trust between schools and families can significantly hinder parental engagement in the Child Find process. Latina mothers have described engaging in meetings in alignment with *respeto:* a cultural value emphasizing respect towards authority. Combined, this cultural tendency of deference, and school teams’ limited efforts to encourage parental input, positioned mothers to merely receive information rather than actively participate in meetings (Mortier and Arias, 2023). Cultural mismatches may also manifest through differing interpretations of disability labels and intervention goals. For example, while many Latino parents value bilingualism as an asset, schools frequently promote English-only interventions, a problem that can be linked to limited bilingual staffing or inadequate resources (Cioè-Peña, 2018). Additionally, some Latino parents perceive special education labels as stigmatizing, which can influence their willingness to consent to further services (Banda et al., 2025; Cioè-Peña, 2018).

Personal levels of acculturation also shape parents’ involvement in their students’ education. Acculturation refers to the ongoing process of cultural and psychological change, where individuals and groups adapt to each other in different ways, resulting in adjustment to the new cultural context while also maintaining aspects of one’s heritage culture. In the context of special education systems, acculturation often involves adapting to a dominant culture, in this case U.S. culture, which may require some Latino parents to navigate language learning, generational differences, and new social norms (Berry, 2005). Within this framework, some individuals may experience assimilation, which refers to adapting to the dominant culture while minimizing or giving up aspects of one’s original cultural identity (Berry, 2005). As a result, some Latino parents may feel pressured to assimilate to United States’ customs and cultural values, sometimes at the expense of their own cultural identity. For instance, Banda et al. (2025) explained that parents face challenges maintaining their traditional values while complying with the norms and expectations of the American school system. Rather than reflecting a lack of engagement, these experiences may instead reflect mismatched expectations. For example, Perrigo et al. (2024) found that Spanish-speaking parents often described parental involvement through *educacion*, which prioritize teaching morals and respectful behavior at home rather than direct school advocacy in school meetings.

Such challenges highlight the tension between maintaining traditional cultural values and adapting to the U.S. educational system. Current federal special education policies emphasize nondiscriminatory evaluation (34C.F.R. § 300.304(c)) but do not provide explicit direction on how schools should incorporate families’ cultural and linguistic values into the IEP decision-making. Although policies cannot specify responses to specific cultural groups, other federal frameworks offer examples of how cultural responsiveness can be embedded into policy expectations and growth in professional practice. For instance, National Association of School Psychologists (2020) standard I.2.4 illustrates how professional expectations can explicitly require school psychologists to respect families’ cultural, linguistic, and experiential backgrounds and to avoid practices that marginalize families. Similarly, the Every Student Succeeds Act (2015) (ESSA) demonstrates how federal policy can require system levels to perform annual evaluations to identify barriers to participation for CLD families, and report strategies to support school-family interactions. Additionally, LEA must allocate funds for professional development for school staff on parent engagement strategies and supports for families [Every Student Succeeds Act, 2015, 20 U.S.C. § 6,318(a)(2)(D)(i)-(iii), (a)(3)(A)–(D)]. Likewise, the CLAS standards by the U.S. Department of Health and Human Services (HHS) provide a model for continuous improvement through ongoing assessment of CLD responsive practices, collecting demographic data to monitor outcomes to inform service delivery. In addition, organizations are expected to partner with the community to design, implement, and evaluate services (U.S. Department of Health and Human Services, Office of Minority Health, 2013). Taken together, these framework examples demonstrate that federal policy can embed cultural responsiveness. Therefore, IDEA could similarly strengthen family engagement by establishing clearer expectations for access and implementation within their federal policy frameworks.

### Systemic factors

Immigration status is another factor that weighs heavily on Latino parents and their decisions to participate in community and school processes. First-generation Latino immigrants often report fears of deportation or family separation, intensified by current national policies (e.g., ending birthright citizenship, American Civil Liberties Union, 2024; Banda et al., 2025). These immigration-related fears and concerns have been documented to reduce parental participation in government assistance programs for which families are otherwise eligible for such as nutrition, health, public safety, and educational services (Trisi and Herrera, 2018). Narratives and survey data further indicate that in communities where immigration enforcement activity is more prevalent, it contributes to adverse mental health and academic outcomes, including elevated dropout rates and behavioral concerns (Gándara et al., 2023; Trisi and Herrera, 2018). Not only does parental participation decrease with immigration related policies, but federal programs such as Title III (ESSA) that provides funding support for immigrant and multilingual families, may also be affected by reductions in current federal funding (EdLaw Center, 2026). Reductions in these resources may limit districts’ capacity to hire bilingual personnel, provide qualified interpreters, and maintain culturally and linguistically responsive supports for immigrant and multilingual families.

The continued presence of these fears within school systems reflects a disconnect between IDEA’s protections and what families experience. The law mandates that *all* children with disabilities are entitled to identification, evaluation, and FAPE protections explicitly designed to protect all families and, in this case, protect families regardless of immigration status (Individuals with Disabilities Education Act, 2004, 20 U.S.C. § 1,412[a][1][A]). More broadly, access to FAPE has been repeatedly challenged in the context of immigration policy; however, courts have consistently rejected efforts to restrict educational access based on immigration status, most notably in Plyler v. Doe (1982). For over three decades, these laws and policies have protected students from being denied public education due to their immigration status. Despite these clear guidelines, challenges have persisted. For example, in 2011 Alabama implemented a law requiring schools to verify the immigration status of newly enrolling students. This law contributed to fear and increased absenteeism (American Immigration Council, 2016). The courts blocked its implementation and have consistently continued to protect students’ rights to access public education. While IDEA clearly guarantees that all children with disabilities have the right to FAPE, this alone is not sufficient to mitigate immigration-related fears. States and individual districts, therefore, have a responsibility to provide clear and accessible information about parental rights in both general and special education settings. When families feel unsafe or uncertain about engaging with schools, the impact is not limited to special education. Districts should prioritize establishing procedures that explicitly protect undocumented families within school settings, and clearly communicate that participation in school processes, including IEP meetings, will not affect, or be affected by immigration status.

Overall, educational professionals should be aware of intersectional barriers that impact Latino parent’s participation in their children’s educational process. Nuanced overlaps of cultural beliefs, immigration status, and social factors (e.g., language barriers, availability & accessibility for school communication and meetings) amalgamate into invisible constraints for many parents. Ultimately, these conjoint barriers illustrate that ethical and technical adherence to IDEA procedures alone does not ensure that families fully understand or can exercise their rights throughout important educational processes.

## Promising practices

Parent training programs are one of the primary strategies currently implemented to improve Latino parent participation, reduce misinformation, bridge the language gap, and provide culturally appropriate resources (Rios and Burke, 2021). While some researchers have been able to develop culturally responsive advocacy programs for increasing knowledge and advocacy skills (Rios and Burke, 2021; Burke et al., 2018) most programs used remain inaccessible due to limited Spanish speaking trained staff. Additionally, while promising, existing parent training programs are also time consuming, and many Latino parents have reported facing challenges in attending school meetings due to work schedules, lack of childcare, and other responsibilities (De La Cruz, 2020). To truly serve Latino families, parent training requires incorporating bilingual materials into existing practices, having qualified translators on hand, employing culturally responsive facilitators, and providing opportunities for peer support meetings that educate families on their parental rights in their predominant language. Other means of supporting families, that do not strictly apply to Latino families and would help promote equity and access across all families, could include using alternative means of communication when reaching out to parents (e.g., text messaging), using remote video services to encourage parent participation in meetings and conversations (e.g., Zoom, Microsoft Teams, Google Meet), and flexible scheduling to accommodate for parents who may have time conflicts that impact their ability to collaborate with schools.

An example of a well-received program is the *Familias Incluidas en Recibiendo Mejor Educación Especial* (FIRME) program. FIRME is an advocacy program aimed to increase Latino parents’ advocacy at schools for their children with Intellectual and other developmental Disabilities. Rios and Burke (2021) developed FIRME using critical race, adult learning, and sociocultural theories to best understand how adults learn, how Latino adults’ characteristics and behaviors may be influenced by their cultural, racial, and ethnic upbringings, and how to use that information to increase individual advocacy on behalf of one’s child with educational disabilities. The program consists of four 3-h long sessions that provide informative, emotional, and skill-building support. Participants are also able to share their own personal experiences. In their effectiveness research, Rios and Burke (2021) found that, after participating in FIRME, Latino parents were significantly more knowledgeable about special education and related services and significantly less distressed. Parents reported an increase in their understanding of special education terms and IDEA policy, and they felt more empowered to participate in their child’s IEP meetings. Parents enjoyed sharing their personal experiences during sessions, fostering relationships with other parents, and found the resources provided by FIRME, such as a special education law and ethics book with supplemental audio resources, helpful (Rios et al., 2021). These findings highlight the potential of culturally responsive training programs to not only increase parental knowledge, but to also foster meaningful participation and advocacy.

Beyond formal training programs, peer support groups have also played a crucial role in empowering Latina mothers while navigating the IEP process. Research has found that, while many mothers felt overwhelmed by IEP meetings due to the overwhelming amount of information, lack of communication, and poor services, they found strength and support in peer groups (Mortier and Arias, 2023). These peer groups formed outside of schools, and from training programs, allowed parents to share their personal experiences in a comfortable and supportive setting (Rios and Burke, 2021).

These findings highlight several areas of improvement among educational administrators, community stakeholders, and lawmakers involved in shaping school policy, particularly when developing inclusive guidelines for CLD populations in special education. Disparities, inconsistencies, and unclear policy language can create confusion for practitioners regarding appropriate next steps when supporting CLD families. In many cases, there is limited guidance about who to consult, when to seek support, and how to navigate complex situations to provide accurate disability identification.

Given their role within the school systems and their professional standards for ethical practice, school psychologists are uniquely positioned to support Latino families as they navigate the special education process. School psychologists can ensure that parents receive clear and accurate information about procedures while also fostering collaborative and trusting relationships between families and schools. Although specific guidance for practitioners working with Latino families remains limited, ethical and professional standards require culturally and linguistically responsive practice (National Association of School Psychologists, 2020).

### Bridging the language gap

As mentioned previously, school psychologists have a professional code to promote family-school collaboration (National Association of School Psychologists, 2020). However, this collaboration is limited when language barriers exist between schools and families. To ensure that parents are active participants in the special education decision making processes and fully understand the information being shared, school psychologists can advocate for the use of interpreters for non-English speakers during the IEP process according to National Association of School Psychologists (2020) Standard II.3.9. Practitioners should also ensure that essential documents are translated into families’ preferred languages and provide clear, accessible explanations of procedural safeguards and IEP rights in the parents’ native language prior to obtaining informed consent per (National Association of School Psychologists, 2020, Standard I.1.3).

To help with this, NASP maintains a directory of bilingual school psychologists by state to support districts in locating linguistically responsive professionals. When bilingual professionals are not available within a district, school psychologists should collaborate with trained interpreters who are familiar with bilingual psychoeducational assessment. In addition, building relationships with family liaisons, community advocates, multilingual community organizations, and agencies serving CLD families can strengthen communication and trust. For example, Liu et al. (2019) recommends contacting neighboring districts with larger populations of speakers of the needed language for recommendations. Additional options include reaching out to professional interpreting agencies as well as consulting local hospitals, county offices, or state interpreter rosters maintained by health departments or judicial branches to identify trained professionals.

The U. S. Department of Education’s Office of Special Education Programs (OSEP) Spanish Glossary of Common IDEA Terms 2nd Edition (SPAN Parent Advocacy Network, 2013) is another valuable resource. Developed through collaboration with experienced translators, family focus groups, and user feedback, the glossary provides standardized translations of commonly used special education terminology across both Part B and Part C of IDEA. Although the glossary does not provide usage guidance, it offers a foundational tool for clarifying unfamiliar IEP terminology and facilitating more effective communication with Spanish-Speaking families.

### Artificial intelligence

As districts consider strategies to strengthen CLD responsive implementation of IDEA, emerging technologies, such as Artificial Intelligence (AI), has the potential to serve as a meaningful supplemental tool for reducing language barriers and helping parents better understand Child Find procedures, including their procedural safeguards and rights. For families navigating complex special education systems in a second language, AI-assisted translation may improve quality of communication. For example, AI can be used as a translator for conveying information for those who need it (University of Illinois Urbana-Champaign, 2024). Recent studies have looked at how AI is being used in practice, and it was found that it saves hours of work per week for many school psychologists (Farmer et al., 2025). However, because the use of AI has only developed recently, there are many existing limitations. It was found that there are many mistakes in the AI engine’s writing style as well as inaccuracies when translating less common languages (University of Illinois Urbana-Champaign, 2024). Moreover, AI has been found to be culturally biased, as five common models demonstrated cultural values similar to English-speaking countries and European countries (Tao et al., 2024).

AI-assisted translations decline in accuracy in more specialized contexts (Alms, 2025). Because of this, even official organizations that have started using AI as a translator—like the Department of Justice—recommend having a human translator check for accuracy. To minimize these limitations, School Psychology Artificial Intelligence (SPAI) was created for school psychologists. SPAI was created to reduce the hours of workload school psychologists have from paperwork each week and to reestablish a human connection from psychologist to student (School Psych AI, 2023). With its *Family Educational Rights and Privacy Act* (FERPA)-compliant platform, psychologists can enter their reports and instantly receive a detailed narrative draft. SPAI also has the option to translate documents, though it needs to be reviewed and edited, but continues to save hours of work. The use of AI cannot compromise student privacy or accuracy of information. Given these considerations, IDEA policy could benefit from more explicit guidance outlining how AI may be ethically and responsibly integrated into special education practices.

The U.S. Department of Education has already issued general guidance on the use of AI in schools (U.S. Department of Education, Office of Educational Technology, 2023). A similar framework tailored specifically to special education would provide more direction for practitioners on how AI tools may be used to enhance family collaborations while maintaining compliance with confidentiality protections. Using the AI-as-a-translator approach could help ensure that parents have a clearer understanding of the information presented to them, thereby strengthening the likelihood that consent is truly informed rather than procedural. Yet, this approach is an emerging practice that requires continuing research. Best practice would continue to utilize established resources such as the ones mentioned above.

### Bridging cultural mismatch

School systems must acknowledge and address the cultural and linguistic barriers that can limit parental participation in the special education process. Domain 8 of the National Association of School Psychologists (2020) Practice Model emphasizes equitable practices for diverse student populations. This responsibility extends beyond recognizing systemic barriers; it requires actively adopting culturally responsive approaches that reduce power imbalances and strengthen trust. The challenge often lies in how these standards are carried out within complex school systems. While school psychologists are ethically committed to culturally responsive practice, time constraints and limited resources make implementation difficult. To support practitioners in this work, several strategies and resources may serve as guidance for strengthening culturally responsive engagement with Latino families. López et al. (2020) recommends embedding services through a cultural framework that acknowledges core Latino cultural values. Incorporating principles such as *respeto, personalismo, familismo, colectivismo spiritualismo* or religion, idioms of distress, and parent structure (e.g., *marianismo* and *machismo*) can strengthen rapport and collaboration. This can be best incorporated and understood if seen through a protective factor lens (Banda et al., 2025; Foxen, 2016).

Banda et al. (2025) provides guidance for mental health professionals on incorporating these cultural values. Practitioners, for example, can incorporate *familismo*, *colectivismo,* and *personalismo*, which amplify the importance of family and community as a major source of support for children by intentionally, and when appropriate, inviting community who are important for the family. For example, practitioners may consider collaborating with trusted community or faith-based organizations, as families may initially seek support within these systems (Banda et al., 2025). In terms of family structure, recognizing that some traditional Latino families may emphasize paternal authority in decision-making, while maternal roles are often centered on caregiving can help guide practitioner interactions. Incorporating *respeto* may involve clearly explaining roles within the special education system while emphasizing that parents are also crucial IEP member. Integrating these considerations into assessment, intervention, and planning can foster culturally affirming service delivery and may reduce cultural mismatch. Yet, while these cultural values are often seen as protective factors, they can also become risk factors depending on family context. For example, strong family connectedness can be a great source of emotional support but can create stress when navigating acculturation, changes in family roles, or family conflict (Foxen, 2016).

For this reason, and more importantly, practitioners must understand that these cultural values do not apply to all Latino families, which is why it is essential to engage in ongoing conversations with families to better understand their individual perspectives, and experiences. This aligns with research emphasizing the importance of adapting services to each family’s cultural context rather than relying on generalized assumptions (Banda et al., 2025). One helpful way to do this is by allowing families to self-identify their racial and/or ethnic identity, rather than placing them into predetermined categories. For example, practitioners can use open-ended questions (e.g., “What is your racial and/or ethnic identity?”) or provide flexible response options (e.g., “Are you…?” with multiple selections and space to write in a response), which allows families to describe their identity in ways that are most meaningful to them (Santiago and Aguayo, 2025; Goforth and Pham, 2021). Practitioners should also be aware that cultural information is intended to better understand a family’s experiences and perspectives, not to determine how well a family has adapted to mainstream U.S. culture. The goal is not to measure assimilation, but rather to understand the cultural values, and experiences that may influence family-school interactions and educational decision-making.

In addition to culturally grounded practice, schools may consider creating structured opportunities for peer connection among parents navigating the IEP process. Peer support groups have been demonstrated to increase empowerment and engagement among Hispanic families (Mortier and Arias, 2023; Rios et al., 2021). These groups can complement practitioners’ efforts by creating shared spaces where families can share experiences, build community, and receive accurate information in accessible formats. Consistent with recommendations from the FIRME program, participation should remain voluntary and facilitated in ways were privacy and confidentiality remain respected. Confidentiality can be achieved following National Association of School Psychologists (2020) guidelines emphasizing respect for dignity and privacy.

Practitioners may also draw from federally funded parent resources. The Center for Parent Information and Resources (Center for Parent Information and Resources, 2013), for example, provides multilingual tools outlining parental rights and advocacy templates. Similarly, the Michigan Alliance for Families (2006), offers updated Spanish-language IDEA and IEP materials. Multimedia resources, such as Spanish-language educational videos developed by Colorín Colorado (2005), can also enhance understanding for families with limited English proficiency. Overall, these tools provide additional support to school psychologists, particularly in districts with limited bilingual staffing.

### Training future professionals

Within the field of school psychology specifically, there is an increasing emphasis on bilingual service delivery and thus on training. This has been promoted by the increasing number of bilingual students that choose a career in school psychology as well as the growing presence of school psychology training with bilingual concentrations. Currently, NASP does not offer specific or national bilingual certification, leaving the onus on states and individual programs to develop their own credentials and documentation of language proficiency. As of 2024, there are eight school psychology graduate programs in the United States that offer formal specializations or optional graduate certificates in bilingualism; importantly, five of these programs are housed at private colleges and four are in New York, severely limiting the accessibility of such programs from students with financial or geographic needs (National Association of School Psychologists, 2024). If culturally and linguistically responsive assessment is a professional expectation, then standardized bilingual training credentialing must be expanded to ensure consistent and equitable service delivery across states.

Overall, to cultivate culturally responsive school climates more broadly, school psychologists may partner with administrators to assess staff training needs related to working with CLD populations. Supporting professional development, drafting inclusive policy statements, and collaborating with cultural liaisons or community partners. These actions build on existing ethical standards rather than expanding the role beyond professional scope.

### Advocacy and systems consultation

Given the documented impact of immigration-related fears on school engagement, school psychologists play a critical role in promoting safety. National Association of School Psychologists (2020) Standard IV.1 emphasizes advocacy for equitable service delivery, including ensuring that services are provided regardless of immigration status. For instance, National Association of School Psychologists (2019) position statement on displaced persons and refugees provides practical guidance on action steps to help the community. Such as, at the systems level, school psychologists may collaborate with administrators to increase transparency and reduce fear-based uncertainty. For example, schools can post clear statements on district websites affirming that all students have the right to education, regardless of immigration status, and outlining confidentiality rights. Administrators can also provide written guidance to staff clarifying district policies regarding immigration-related questions and the protection of student records.

For currently enrolled students, school psychologists can consult with district leadership to develop step-by-step procedures for responding to requests from immigration enforcement agencies, ensuring that all actions align with legal requirements and district policy. In situations where families express fear, practitioners can provide psychoeducation regarding student rights, refer families to appropriate legal or community resources, and support the development of family preparedness plans when appropriate. Additionally, if immigration enforcement activity occurs near school communities, school psychologists can collaborate with administrators to communicate reassurance to families, clarify rights, and provide counseling supports to students experiencing stress or fear. Such efforts reinforce that schools remain safe and supportive environments.

To cultivate culturally responsive school climates more broadly, school psychologists may partner with administrators to assess staff training needs related to working with CLD populations. Such assessments can help identify gaps in cultural awareness and confidence when working with Latino families. Similar to the CLAS standards by HHS, assessment results can be used to guide targeted professional development efforts and monitor progress over time.

Based on identified needs, school psychologists and system-level professionals can collaborate with cultural liaisons, community organizations, and family stakeholders to develop learning opportunities tailored to the cultural contexts of their school communities. Professional development may include education regarding cultural assets commonly identified within Latino communities, such as familismo, respeto, personalismo, colectivismo, spiritual beliefs, and family role structures. Increasing awareness of these cultural values may help educators better understand how families approach communication, trust-building, decision-making, and school involvement. This understanding can support more culturally responsive practices and foster more meaningful family participation in educational decision-making processes. These actions build on existing ethical standards rather than expanding the role beyond professional scope.

## Moving forward collaboratively

At its core, this review underscores a critical tension within special education systems: while policies such as IDEA establish a framework for access, access alone does not guarantee equity. To promote equitable participation for Latino families in special education, policy action must include clear guidelines for working with Spanish-speaking Latino families. Latino families continue to navigate systems in which participation is technically permitted but functionally constrained by linguistic, cultural, and systemic barriers. Meaningful participation requires more than procedural adherence, it demands intentional, culturally responsive implementation that centers understanding, trust, and collaboration. Although IDEA requires meaningful access to information, it does not specify which “vital documents” must be translated or when. This lack of clarity results in inconsistent implementation across districts. Parents have the right to request interpreters and translated materials; however, many are unaware of these rights or how to exercise them. While all families receive a Procedural Safeguards Notice, although not always in the preferred language, the document’s legal and technical language can make it difficult for many parents to understand their rights. Looking forward, there is a critical opportunity to reimagine how access is operationalized within special education systems by leveraging interdisciplinary approaches, emerging tools, and culturally grounded practices to reduce long-standing barriers.

School psychologists are not alone in their ethical and professional mandate to work towards more equitable education practices for all. School administrators, community stakeholders, school board members, and educational lawmakers should be informed and compelled toward making systemic and local changes for more equitable practice. To address these critical concerns, several actions could occur. School districts and state agencies should develop parent-friendly procedures and rights packages that summarize key protections that are accessible in families’ preferred languages. Districts can also promote equity by partnering with organizations, such as the Center for Parent Information and Resources, that can help with bilingual resources. Administrators, teachers, school board members, policymakers, and community partners may benefit from professional learning opportunities that emphasize understandings of familismo, colectivismo, respeto, personalismo, spiritual beliefs, and family role structures. Such training can help stakeholders recognize how these cultural values may influence communication preferences. Finally, districts must establish transparent procedures to protect undocumented parents and reaffirm that all children, regardless of immigration status, and have FAPE rights.

Collectively, these practices can increase equity, transparency, and trust, which align with the ethical principles of informed consent, fairness, and social justice that are foundational for school-based service providers. Moving forward, policymakers, practitioners, and training programs must shift from compliance-driven models to equity-driven practices that operationalize access in ways that are truly inclusive. Without this shift, the promise of IDEA remains only partially fulfilled. Ultimately, this review seeks to contribute to the field by centering the lived experiences of Latino families within special education and highlighting the gap between policy intent and practice. It is the hope that this work not only informs future research and policy development but also encourages practitioners to critically reflect on their role in shaping more inclusive and collaborative special education systems for Latino families.

## Footnotes

1 The language used to describe identity in this article reflects an effort to center culturally responsive practices in school psychology. The term *Latino* is used to refer to individuals with origins in Latin America and the Caribbean. The term *Hispanic* is used to refer to individuals from Spanish-speaking countries. The term *Latinx/e* is used to acknowledge gender-inclusive identities. Given the wide range of terms used to describe this population, it is important to recognize that no single label captures all identities or preferences. As a result, these terms may be used interchangeably when appropriate. When referring to the population more broadly, terms such as *Latino* or *Hispanic* are often used, while also acknowledging that individuals may identify differently based on personal factors (García, 2020).
